# Pulmonary cyst newly formed after lobectomy in various underlying lung conditions

**DOI:** 10.1186/s40792-024-01861-6

**Published:** 2024-03-11

**Authors:** Satona Tanaka, Naoki Date, Yoshito Imamura, Takafumi Kabuto, Hidenao Kayawake, Hideki Motoyama, Akihiro Aoyama, Hirohi Date

**Affiliations:** 1 Department of Thoracic Surgery, Kyoto University Hospital, 54, Shogoin-Kawahara-Cho, Sakyo-Ku, Kyoto, 606-8507 Japan; 2 Department of Thoracic Surgery, Nagoya University Hospital, 65, Tsurumai-Cho, Showa-Ku, Nagoya, 466-0065 Japan; 3 Department of Thoracic Surgery, Japanese Red Cross Wakayama Medical Center, 4,20 Komatsubaradori, Wakayama, 640-8558 Japan; 4 Department of Thoracic Surgery, Kobe City Medical Center General Hospital, 2,1,1 Minatojimaminamimachi, Chuo-Ku, Kobe, 650-0047 Japan; 5 Department of Thoracic Surgery, Kobe City Nishi-Kobe Medical Center, 5,7,1 Kojidai, Nishi-Ku, Kobe, 651-2273 Japan; 6 Department of Thoracic Surgery, Kyoto Katsura Hospital, 17, Yamadahirao-Cho, Nishikyo-Ku, Kyoto, 615-8256 Japan

**Keywords:** Pulmonary cyst, Pneumatocele, Lobectomy

## Abstract

**Background:**

It has been recently recognized that pulmonary cyst may develop after pulmonary resection, causing various symptoms. Most previously reported cases were after upper lobectomy in patients with chronic obstructive lung disease (COPD).

**Case presentation:**

Case 1 was a man in his 70 s with interstitial pneumonia (IP). Right lower lobectomy was performed for metastatic lung tumor using video-assisted thoracoscopic surgery (VATS). On postoperative day (POD) 19, computed tomography (CT) revealed a large cyst at the upper interlobular surface of the middle lobe, with pneumoderma and pneumomediastinum. The cyst was incised, polyglycolic acid (PGA) sheet and fibrin glue were applied, and the cyst was sutured. The sutured line was covered again with PGA sheet and fibrin glue. Case 2 was a man in his 70 s with COPD. Right upper lobectomy for primary lung cancer was performed using VATS. On POD 17, CT revealed a large pulmonary cyst at the apex of S6 and massive air leakage was observed. The same surgical procedure as that used in case 1 was performed. Cases 3 and 4 were healthy donors for living-donor lung transplantation. Two months after the right lower lobectomy in Case 3 and 3 months after the left lower lobectomy in Case 4, the patients had respiratory symptoms such as dyspnea and hemosputum. CT revealed a large cyst on the diaphragmatic surface of the right middle lobe in Case 3 and on the posterior mediastinal surface of the left upper lobe in Case 4. Cyst incision, soft coagulation, and application of PGA sheet with fibrin glue were performed in both cases. CT performed 1 year after surgery showed no development of a pulmonary cyst or air space in these four cases.

**Conclusions:**

Pulmonary cysts newly formed after lobectomy can develop not only in COPD or IP but also in healthy lungs. Our findings suggest that incision of the cyst and application of fibrin glue and PGA sheet with or without suturing the cyst wall is effective for management.

## Background

It has recently been reported that pulmonary cysts or pneumatoceles may develop early after pulmonary resection. Newly formed pulmonary cysts cause various respiratory symptoms, including prolonged postoperative air leakage, pneumothorax, subcutaneous emphysema, pneumomediastinum, and hemosputum [1–7]. These complications are believed to result from dissection between the fragile pleura and lung parenchyma and are considered to occur in patients with lung diseases such as chronic obstructive pulmonary disease (COPD). They developed after upper lobectomies in most cases [1–7]. Herein, we describe four cases of newly developed pulmonary cysts after lobectomy not only in patients with lung disease but also in healthy patients.

## Case presentation

### Case 1

**Fig. 1:**
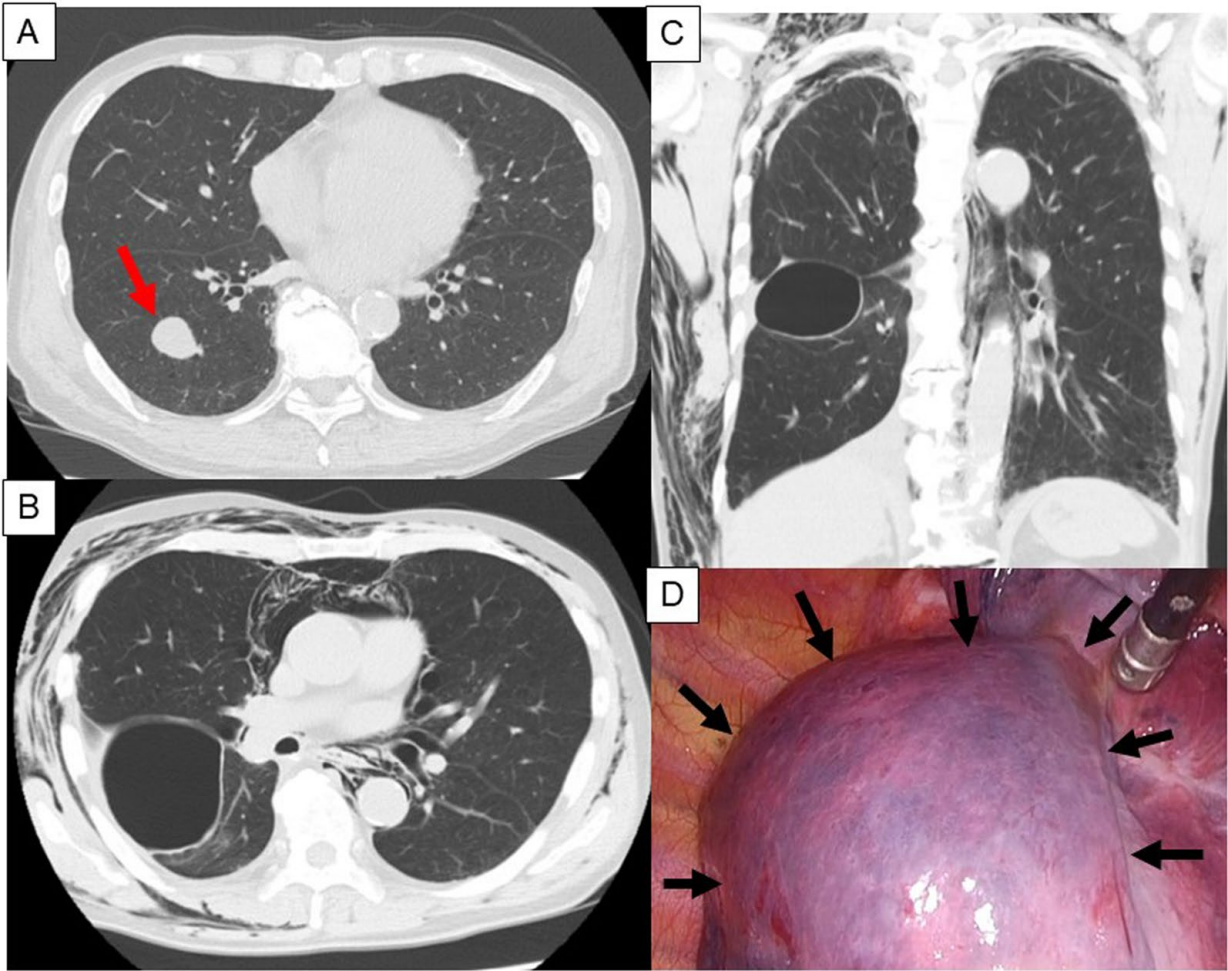
Preoperative CT and newly formed pulmonary cyst in Case 1. Preoperative CT showing a nodule of 23 mm in the middle of the right lower lobe (arrow). CT reveals few fibrotic changes; however, the patient was diagnosed with interstitial pneumonia by histopathology of the resected lung (**A**). Postoperative CT performed on POD 17 shows a newly developed pulmonary cyst at the upper interlobular surface of the middle lobe with subcutaneous emphysema and pneumomediastinum (axial section: **B**, coronal section: **C**). A broad-based pulmonary cyst is observed during reoperation (arrows) (**D**)

**Fig. 2:**
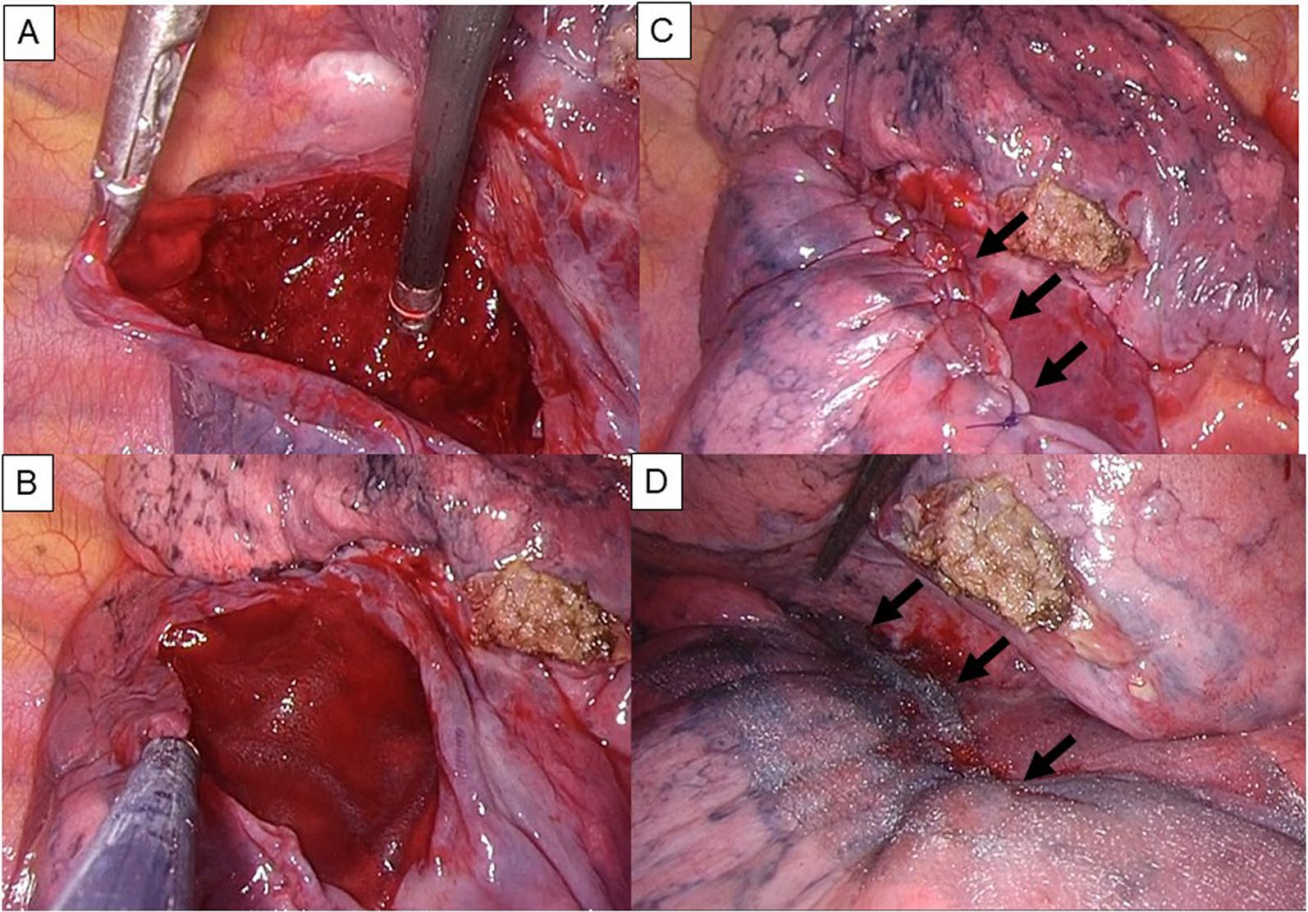
Operative procedure in Case 1. The cyst wall is incised (**A**), and PGA sheet is applied on the base of the cyst with fibrin glue (**B**). Then, the cyst wall is sutured (arrows) (**C**) and the suture line is covered again with PGA sheet and fibrin glue (arrows) (**D**)

A man in his 70 s underwent right lower lobectomy using uniportal video-assisted thoracoscopic surgery (VATS) for a solitary metastatic lung tumor from undifferentiated pleomorphic sarcoma of the lower extremity (Fig. 1A). Smoking history was 43 pack-years. The preoperative pulmonary function test (PFT) demonstrated forced vital capacity (FVC) was 4050 mL (%FVC 113.1%), and the ratio of forced expiratory volume in 1 s to FVC (FEV1.0%) was 77.5%. The lung was completely lobulated, and we did not need to use staplers to divide the fissure. The intraoperative sealing test revealed no air leakage. The pathological diagnosis was metastasis of undifferentiated pleomorphic sarcoma with interstitial pneumonia that was preoperatively undiagnosed using computed tomography (CT). The patient was discharged on postoperative day (POD) 6. The patient was re-admitted with subcutaneous emphysema and pneumomediastinum on POD 19. Computed tomography (CT) revealed a newly developed large pulmonary cyst in the middle lobe at the interlobar plane between the middle and upper lobes (Fig. 1B, C). No pneumothorax was observed. VATS was performed on POD 20. A pulmonary cyst was noted at the interlobar surface of the middle lobe, between the middle and upper lobes. (Fig. 1D). Blood clots and multiple air leakages were detected inside the cysts (Fig. 2A). After incision of the cyst wall, a polyglycolic acid (PGA) sheet and fibrin glue were applied inside the cyst (Fig. 2B). The cyst wall was sutured using a 4-0 absorbable monofilament suture (Fig. 2C) and covered again with PGA sheet and fibrin glue (Fig. 2D). Postoperative course was uneventful. CT 1 year after surgery showed no development of a pulmonary cyst or air space.

### Case 2

**Fig. 3:**
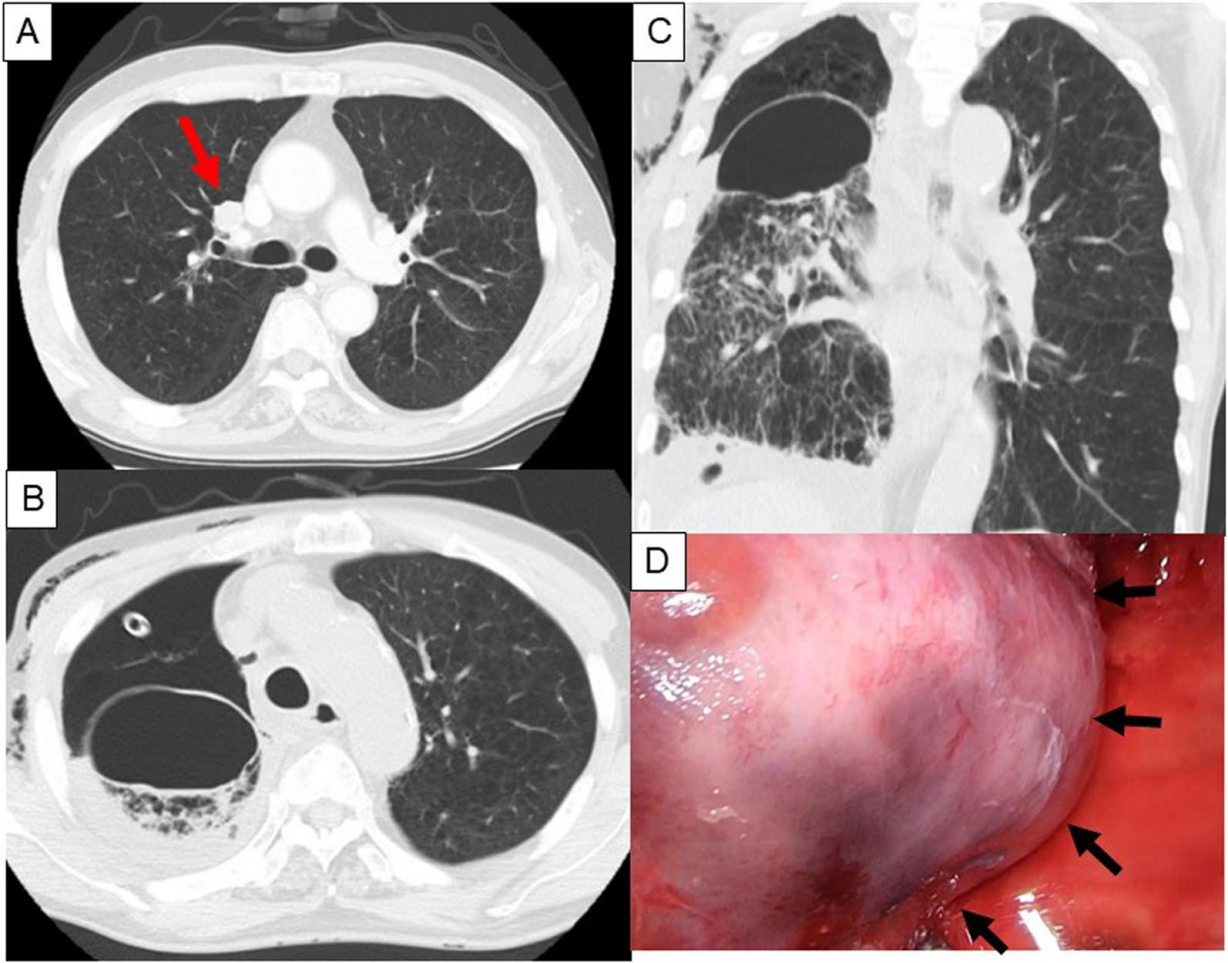
Preoperative CT and newly formed pulmonary cyst in Case 2. Preoperative CT shows a nodule measuring 16 mm in the right S3 (arrow) and severe emphysematous changes (**A**). Postoperative CT performed on POD 17 reveals newly developed pulmonary cyst at apex of S6 (axial section: **B**, coronal section: **C**). Broad-based pulmonary cyst with hole is recognized during reoperation (arrows) (**D**)

A man in his 70 s with COPD underwent right upper lobectomy with mediastinal lymph node dissection using multi-portal VATS for a right S3 nodule that was diagnosed as primary lung cancer (pathological stageIA2) (Fig. 3A). Smoking history was 52 pack-years. Preoperative PFT demonstrated FVC of 3193 mL (%FVC 120.6%) and FEV1.0% was 35.9%. CT revealed severe emphysematous changes. The interlobular planes between the upper and middle lobes, and between the upper and lower lobes, were divided using a stapler. Intraoperative sealing test revealed air leakage in S6 after interlobular dissection, which was sutured and covered with a PGA sheet and fibrin glue. Postoperative air leakage was observed and pleurodesis was repeated, resulting in decreased air leakage. However, massive air leakage was observed on POD 17. CT revealed a newly developed large pulmonary cyst at the apex of S6 (Fig. 3B, C). On POD 19, open thoracotomy was performed. The pulmonary cyst was observed at the apex of S6, and its location was different from that of the sutured part during the first surgery (Fig. 3D). Massive air leakage was observed in the pulmonary cyst hole. Blood clots and multiple air leakages were observed inside the cyst. The procedure was the same as that for Case 1. Postoperative course was uneventful. CT 1 year after surgery showed no development of a pulmonary cyst or air space.

### Case 3

**Fig. 4:**
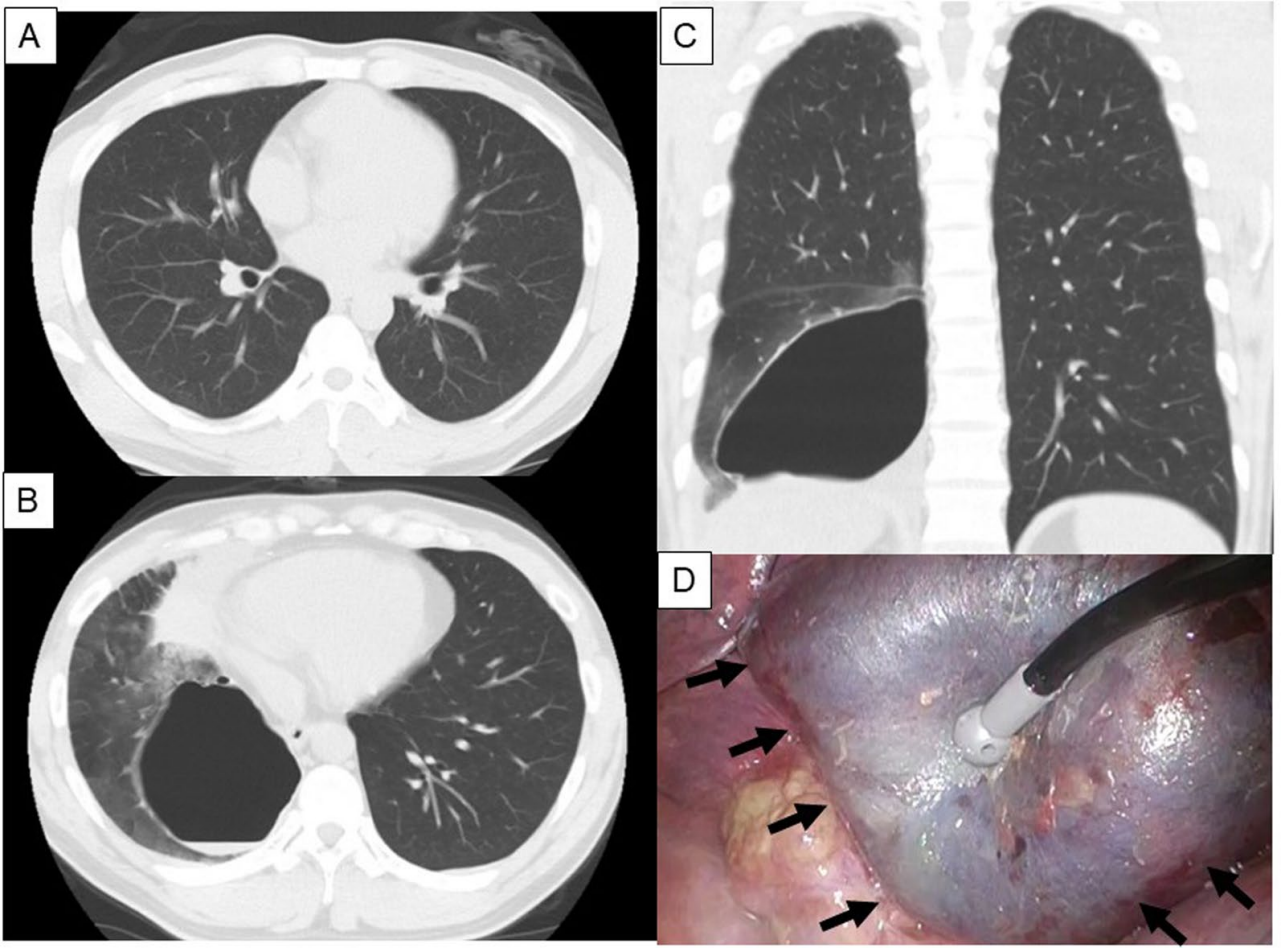
Preoperative CT and newly formed pulmonary cyst in Case 3. Preoperative CT shows normal lung (**A**). Postoperative CT performed 2 months after surgery reveals newly developed pulmonary cyst at diaphragmatic surface of middle lobe with some fluid inside the cyst (axial section: **B**, coronal section: **C**). Broad-based huge pulmonary cyst is recognized at re-operation (arrows) (**D**)

**Fig. 5:**
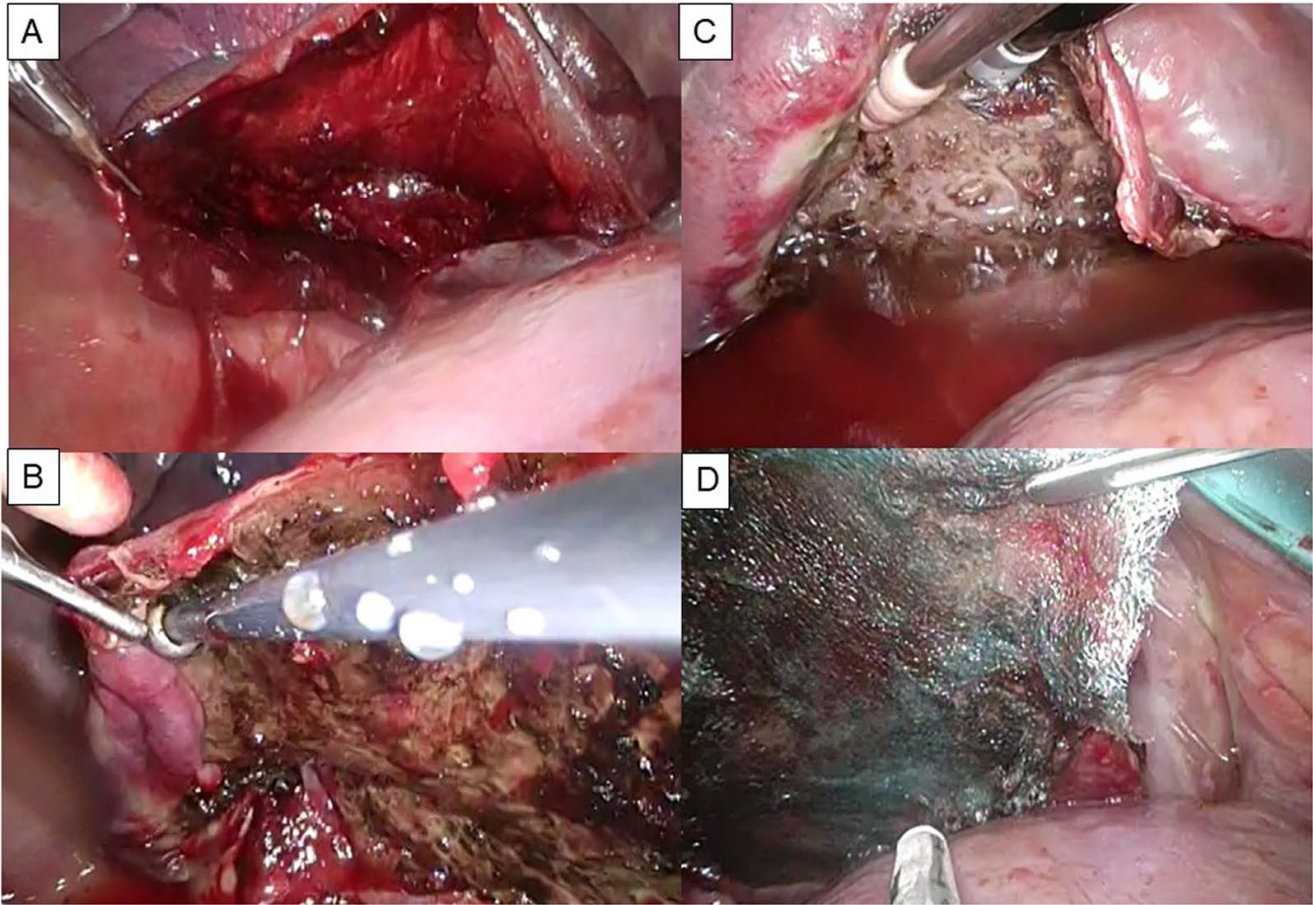
Operative procedure in Case 3. The cyst is incised and the blood clot is removed (**A**). Soft coagulation is applied (**B**), and sealing test reveals mild air leakage (**C**). PGA sheet is applied with fibrin glue to the surface of the base of the cyst without suturing (**D**)

A man in his 30 s without any medical history underwent right lower lobectomy through posterolateral thoracotomy as a living donor for lung transplantation (Fig. 4A). Smoking history was five pack-years. Preoperative PFT demonstrated FVC of 4320 mL (%FVC 92.9%), and FEV1.0% was 83.3%. The interlobular plane between the upper and lower lobes was divided by using a stapler. The middle and lower lobes were completely lobulated, and a stapler was not used to divide the fissure. The intraoperative sealing test revealed no air leakage. The patient was discharged on POD 7. He sneezed loudly because of a seasonal allergy after discharge and was re-admitted with cough and hemosputum 2 months after surgery. CT revealed a newly developed huge pulmonary cyst at the diaphragmatic surface of the middle lobe, with fluid inside the cyst (Fig. 4B, C). VATS was performed. Broad-based cystic lesion was observed on the diaphragmatic surface of the middle lobe (Fig. 4D). After the cyst incision, blood clots and multiple air leaks were observed (Fig. 5A). A PGA sheet and fibrin glue were applied after monopolar hemostatic soft coagulation at the broad base of the cyst (Fig. 5B). The cyst wall was not sutured because air leakage was not severe (Fig. 5C, D), and the base of the cyst in the middle lobe was so large that suturing might cause volume reduction of the middle lobe. Postoperative course was uneventful. CT 1 year after surgery showed no development of a pulmonary cyst or air space.

### Case 4

**Fig. 6:**
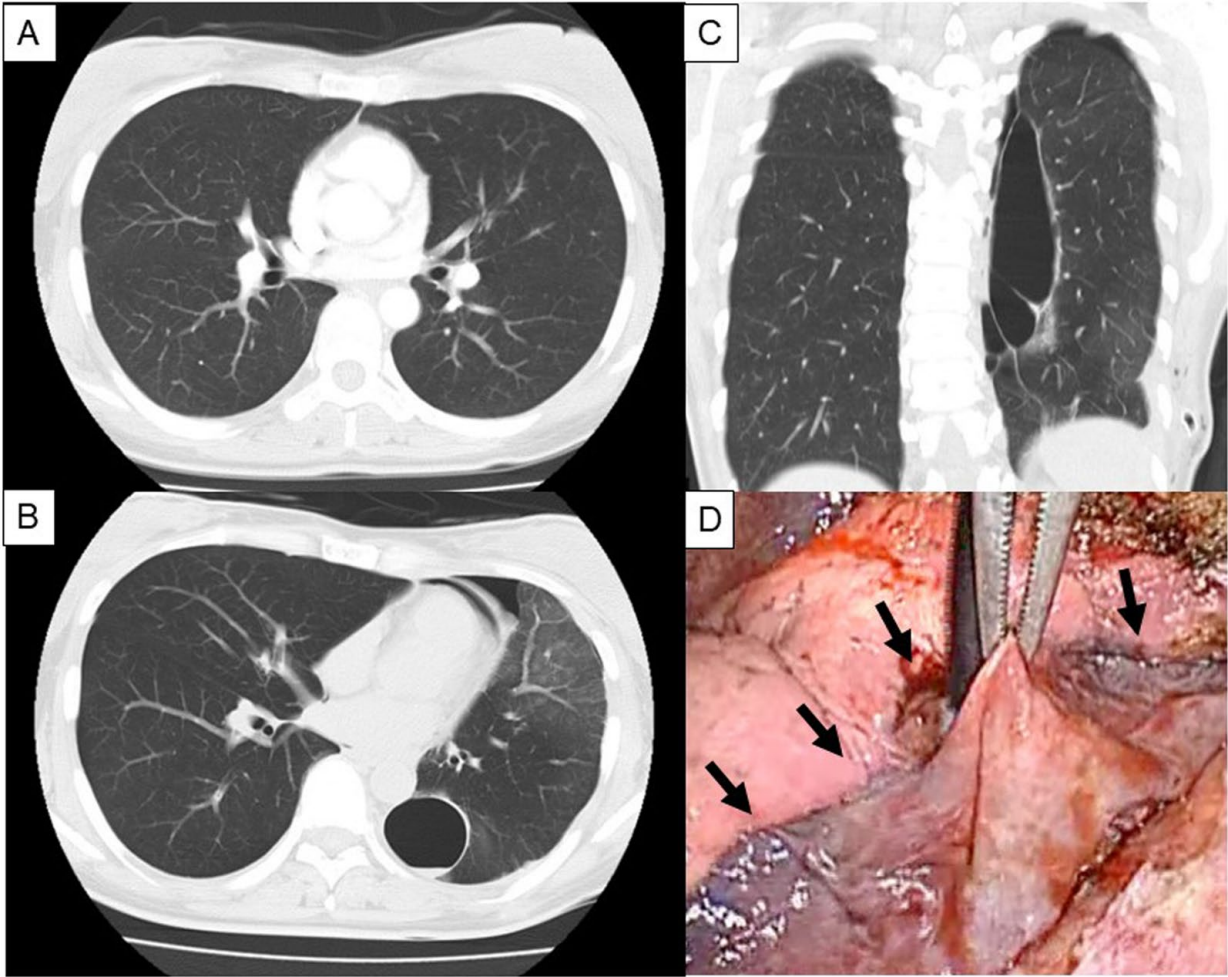
Preoperative CT and newly formed pulmonary cyst in Case 4. Preoperative CT shows a normal lung. (**A**). Postoperative CT performed 3 months after surgery reveals a newly developed pulmonary cyst at the posterior mediastinal surface of left upper lobe with ground glass opacity in left upper lobe (Axial section: **B**, Coronal section: **C**). Broad-based longitudinally huge pulmonary cyst with tear is recognized at re-operation (arrows) (**D**)

A woman in her 30 s without any past medical or smoking history underwent left lower lobectomy through posterolateral thoracotomy as a living donor for lung transplantation (Fig. 6A). Preoperative PFT demonstrated FVC of 5320 mL (%FVC 165.7%), and FEV1.0% was 77.44%. The lung was completely lobulated, and we did not need to use staplers to divide the fissure. An intraoperative sealing test revealed no air leakage. The patient was discharged on POD 13. She was a clarinet player, and we allowed her to resume playing the clarinet one month after the surgery, and she was re-admitted with cough and dyspnea 3 months after surgery. CT revealed a newly developed, longitudinally large pulmonary cyst at the posterior mediastinal surface of the left upper lobe, with fluid inside the cyst (Fig. 6B, C). Re-thoracotomy was performed. A broad-based, longitudinally large cystic lesion was noted with adhesion on the posterior mediastinal side of the left upper lobe. The cyst wall was torn (Fig. 6D). Blood clots and multiple air leakages were observed inside the cyst. The procedure was the same as that for Case 3. The cyst wall was not sutured in this case either, because the base of the cyst in the left upper lobe was so large that suturing might cause volume reduction. Postoperative course was uneventful. CT 1 year after surgery showed no development of a pulmonary cyst or air space.

## Discussion

Here, we report four cases of pulmonary cysts that newly developed after lobectomy. Four patients had respiratory symptoms, such as air leakage, dyspnea, subcutaneous emphysema, pneumomediastinum, and hemosputum. They had different underlying lung conditions, including COPD, IP, and donation of a healthy lung for living-donor lobar lung transplantation. They were successfully managed by the cyst incision and the application of a PGA sheet and fibrin glue, with or without suturing the cyst wall. The treatments were considered appropriate because all cases showed no cyst or pleural space on chest CT 1 year after surgery. A summary of our cases and previously reported cases is shown in Table 1.

**Table 1:** Cases with pulmonary cysts newly developed after lobectomy and segmentectomy

Case	Author	Age	Sex	Underlying lung condition	Resected lung at initial surgery	Recognition of cyst	Location of cyst
A	Sugimura et al	80	Male	COPD	LUL	Immediately in OR	LLL (S6)
B	Kawamoto et al	49	Male	COPD	RUL	10 days	RLL (S6)
C	Fujibayashi et al.	69	Male	Asthma, CPAP for SAS	RUL	29 days	RLL (dorsal surface)
D	Case 1*	70 s	Male	IP	RLL	19 days	RML (interlobular surface adjacent to RUL)
E	Case 2*	70 s	Male	COPD	RUL	17 days	RLL (S6)
F	Honma et al.	74	Male	Unknown	Upper segment of LUL	4 days	LLL (diaphragmatic surface)
G	Yoo et al.	62	Male	Unknown	LUL	9 days	LLL (S6)
H	Kondo et al.	56	Male	Healthy	RUL	1 month	RLL (diaphragmatic surface)
I	Kondo et al.	57	Male	Healthy	LUL	9 months	LLL (diaphragmatic surface)
J	Case 3*	30 s	Male	Healthy	RLL	2 months	RML (diaphragmatic surface)
K	Case 4*	30 s	Female	Healthy	LLL	3 months	LUL (mediastinal surface)

*Cases 1, 2, 3, and 4 are included in this report

*COPD* chronic obstructive pulmonary disease, *CPAP* continuous positive airway pressure, *IP* interstitial pneumonia, *LLL* left lower lobe, *LUL* left upper lobe, *OR* operating room, *RLL* right lower lobe, *RML* right middle lobe, *RUL* right upper lobe, *SAS* sleep apnea syndrome, *S6* segment 6 (superior segment of the lower lobe)

This complication has been reported to cause prolonged or massive postoperative air leakage, hemosputum, pneumothorax, subcutaneous emphysema, and pneumomediastinum after pulmonary resection. In previously reported cases, about half of the patients had COPD, and the initial procedures were right and left upper lobectomies and segmentectomy of the left upper lobe. The location of the cyst was limited to the surface of the remaining lung which was not adjacent to other lobes such as the apex and diaphragmatic surface. The timing of the recognition of pulmonary cysts ranged from immediately after surgery to 9 months after surgery [1–7]. Most patients were diagnosed with this condition by chest CT; however, Sugimura et al. reported that they diagnosed the patient immediately after left upper lobectomy while the patient was still in the operating room [2]. They performed a reoperation due to massive air leakage and found cyst formation in the superior segment of the left lower lobe. This case series is the first to describe a pulmonary cyst that developed not only after resection of the upper lobe but also after lower lobectomy, and that the location of the cyst was not only at the apex and diaphragmatic surface but also at the interlobular and mediastinal surfaces.

The detailed mechanism of cyst formation is still unknown; however, several reports have described the pathological findings of resected cysts [4–7]. In these reports, the cyst wall was found to contain visceral pleura and alveolar cells, indicating that subpleural dissection occurred following the initial pulmonary resection. This dissection may have been caused by increased intrathoracic negative pressure after lung resection or by increased intrapulmonary positive pressure resulting from the patients’ activities and check valve mechanism due to flexion of the airway after relocating the remnant lung. While surgical manipulation of the remaining lung may be associated with this dissection, it does not fully explain the occurrence of such cysts because we experienced cyst formation at the interlobar plane between the middle and upper lobes of the remaining middle lobe, and a previous study reported cyst formation on the diaphragmatic surface after upper lobectomy [5]. These areas should be intact during the initial lung resection.

In our experience, this complication occurred both in patients with underlying lung disease, such as COPD (Case 1) and interstitial pneumonia (Case 2), and in those without any underlying lung disease (Cases 3 and 4). In cases 1 and 2, the main reason for subpleural dissection might be the underlying lung disease. The location of the cyst in Cases 1 and 2 was not manipulated during the initial surgery. Cases 3 and 4 were healthy donors for living-donor lung transplantation, and it was speculated that sneezing due to seasonal allergy and playing the clarinet might have caused the frequent and rapid increase in intrapulmonary positive pressure, which led to cyst formation. Cases 3 and 4 are similar to those described by Kondo Y et al. in terms of the recognition of cysts later after surgery in a healthy lung [5]. Based on our experiences and the reported cases, newly formed pulmonary cysts after lobectomy might be classified into two groups. One is the cyst formed in patients with lung disease such as COPD, where fragile lung tissue due can be a reason of subpleural dissection and the cyst tends to develop early after the initial surgery (Cases A to E in Table 1). The other is the cyst developed in healthy lungs, where high intrapulmonary positive pressure or intrathoracic negative pressure can be a reason, and the cyst tends to develop later after the initial surgery (Cases H to K in Table 1).

In terms of surgical management, we followed the procedure for all reported cases. Kondo et al. reported the management of a similar case to Case 3 and Case 4 in our series, where the cyst wall resection, soft coagulation of the bottom of the cavity, and covering with a fibrin sealant patch were performed [5]. The necessity of suturing the cyst wall should be determined based on the size of the cavity and severity of air leakage. CT performed 1 year after surgery revealed no recurrence of the cyst, and the outcomes of our procedures were satisfactory.

## Conclusions

New development of pulmonary cysts, which cause hemosputum, pneumothorax, subcutaneous emphysema, and pneumomediastinum, occurred not only in patients with lung diseases such as COPD, but also in healthy patients. The cyst developed after lower lobectomies as well as upper lobectomies. Surgical management consisting of incision of the cyst and application of PGA sheet and fibrin glue with or without suturing the cyst wall was effective.

## Data Availability

Not applicable.

## Abbreviations

COPD: Chronic obstructive lung disease
CPAP: Continuous positive airway pressure
CT: Computed tomography
FEV1.0%: The ratio of forced expiratory volume in 1 s to forced vital capacity
FVC: Forced vital capacity
IP: Interstitial pneumonia
LLL: Left lower lobe
LUL: Left upper lobe
OR: Operation room
PFT: Pulmonary function test
PGA: Polyglycolic acid
POD: Postoperative day
RLL: Right lower lobe
RML: Right middle lobe
RUL: Right upper lobe
SAS: Sleep apnea syndrome
S6: Segment 6 (superior segment of lower lobe)
VATS: Video-assisted thoracoscopic surgery
